# Neuroinflammation Treatment via Targeted Delivery of Nanoparticles

**DOI:** 10.3389/fncel.2020.576037

**Published:** 2020-09-30

**Authors:** Susana R. Cerqueira, Nagi G. Ayad, Jae K. Lee

**Affiliations:** ^1^Department of Neurological Surgery, Miller School of Medicine, University of Miami, Miami, FL, United States; ^2^The Miami Project to Cure Paralysis, Miller School of Medicine, University of Miami, Miami, FL, United States; ^3^Sylvester Comprehensive Cancer Center, Miller School of Medicine, University of Miami, Miami, FL, United States

**Keywords:** nanoparticles, drug delivery, multifunctional nanoparticles, CNS, neuroinflammation, theranostics, stimuli-responsive, cell targeting

## Abstract

The lack of effective treatments for most neurological diseases has prompted the search for novel therapeutic options. Interestingly, neuroinflammation is emerging as a common feature to target in most CNS pathologies. Recent studies suggest that targeted delivery of small molecules to reduce neuroinflammation can be beneficial. However, suboptimal drug delivery to the CNS is a major barrier to modulate inflammation because neurotherapeutic compounds are currently being delivered systemically without spatial or temporal control. Emerging nanomaterial technologies are providing promising and superior tools to effectively access neuropathological tissue in a controlled manner. Here we highlight recent advances in nanomaterial technologies for drug delivery to the CNS. We propose that state-of-the-art nanoparticle drug delivery platforms can significantly impact local CNS bioavailability of pharmacological compounds and treat neurological diseases.

## Introduction

Neurological disorders disrupt the normal function of the brain and/or spinal cord and are a major cause of death and disability worldwide. Dysfunction in the central nervous system (CNS) leads to variable levels of impairments in speech, memory, sensorimotor, and autonomic functions, which can considerably diminish the patients’ quality of life. The underlying causes of neurological diseases are often complex and diverse, and can stem from degeneration, trauma, infection, tumors, vascular dysfunction, structural defects, or autoimmune conditions (Feigin et al., 2019). Recent insights into the mechanisms underlying several neurological disorders are opening new windows of opportunity to advance preclinical findings into clinical options. However, successful translation is still challenging in many different CNS pathologies and therefore treatments remain limited. Accumulating evidence suggests neuroinflammation as a common feature of virtually every neurological disorder, whether as a primary driver of disease or as a response to neurological dysfunction (Gilhus and Deuschl, 2019). Pharmacological inhibition of inflammation has induced neuroprotection in several preclinical studies in the CNS (Glass et al., 2010; Azodi and Jacobson, 2016). Therefore, targeting cells and molecular pathways that contribute to neuroinflammation has the potential to become a successful disease-modifying strategy for the treatment of most neurological disorders.

The term neuroinflammation has been used to describe different cellular and molecular pathological phenomena that can encompass activation of CNS-resident cells, CNS infiltration by circulating leukocytes, and production of inflammatory mediators in the CNS environment (Waisman et al., 2015; Ransohoff, 2016). Microglia and astrocytes are the main CNS cell types actively involved in the immune response. In addition, under certain pathological conditions, peripheral circulating lymphocytes, neutrophils, and monocytes can infiltrate the CNS and exacerbate the neuroinflammatory response. When activated, both resident and infiltrating cells are responsible for the production of inflammatory mediators, particularly cytokines, which are key molecular players in inflammation. One common feature of most cytokines is their pleiotropic nature. The same cytokine can trigger an inflammatory cascade that can have detrimental or protective functions in disease progression depending on timing, area of action, or cellular source (Becher et al., 2017; Brambilla, 2019). For example, tumor necrosis factor (TNF) is one of the most widely studied cytokines in stroke, and preclinical data indicates both neurotoxic and neuroprotective effects of TNF. Genetic studies indicated neuroprotective effects for microglial-derived TNF in murine ischemic models (Lambertsen et al., 2009). However, TNF was also shown to contribute to endothelial cell necroptosis and vascular leakage that lead to worsened ischemic brain injury (Chen et al., 2019). Given the complexity of cellular and molecular phenomena involved in neuroinflammation, cell targeted pharmacological approaches seem to be an essential component in the design of effective treatments. While technological progress in biochemical, genetic, and imaging analysis are allowing significant advances in the understanding of the pathobiology of disease, effective targeted drug delivery options for CNS remain elusive.

Spinal cord injury (SCI) involves a complex time-dependent course of cellular and molecular events that have been elucidated in the last several decades. An SCI induces vascular damage and ischemia that lead to cell necrosis and apoptosis, followed by acute and chronic neuroinflammation, glial activation, scar formation, and demyelination and neurodegeneration (O’Shea et al., 2017; Tran et al., 2018). These events result in overall regenerative failure and can become highly debilitating. Despite abundant preclinical data supporting anti-inflammatory treatments in SCI, clinical trials evaluating safety and efficacy of anti-inflammatory drug administration have failed (Badhiwala et al., 2018). The failure in clinical translation can be due to several factors, among them ineffective transport of drug compounds to relevant CNS targets at therapeutic concentrations.

Nanotechnology advances have recently yielded countless innovative biomaterials that can be custom designed and serve as drug delivery systems to effectively target biological tissues with cell selectivity. The majority of nanoparticle-based therapeutics has focused on targeting tumor cells and provide enhanced antitumor efficacy. Many of these technologies are now undergoing clinical evaluation, and some have been approved clinically to treat a variety of cancer diseases (Wolfram and Ferrari, 2019). The vast body of work on tumor-specific nanoparticle drug delivery has been extensively reviewed elsewhere (van der Meel et al., 2019; Guo et al., 2020). In this review, we will focus on exploring how emerging nanomaterials can help overcome the current limitations of drug delivery to the CNS, giving particular attention to the development and applications of cell-targeted and stimuli-responsive nanoparticles to modulate neuroinflammation. Advanced nanoparticles that deliver bioactive compounds to CNS intracellular targets at optimal doses, and in a spatiotemporal controlled manner can finally yield treatment options for neurological disease.

## Challenges and Options in Drug Delivery to Treat Neuroinflammation

The ideal delivery of a pharmacological anti-inflammatory agent to the CNS entails non-invasive administration of a stable compound at a therapeutic concentration followed by rapid targeting of dysfunctional pathways, and reversal of the disease to a healthy state without induction of off-target effects. However, in the case of drug delivery to the CNS we encounter several roadblocks that complicate effective targeting. First, most non-invasive routes of delivery are ineffective in providing access to the CNS, due to the distinctive properties of the neurovascular unit that restricts most pharmacological compounds from entering the CNS (Pandit et al., 2019). In addition, most bioactive therapeutic small molecule compounds that are able to enter the CNS are lipophilic drugs that have compromised stability and short half-lives in physiological environments, leading to challenges in maintaining therapeutic concentrations (Gribkoff and Kaczmarek, 2017). Finally, conventional drug delivery strategies lead to widespread drug diffusion in the organism causing undesired off-target effects. For instance, corticosteroids are used regularly in the clinic as anti-inflammatory and immunosuppressive drugs to manage demyelinating disorders, infections, and neurotrauma. Corticosteroids exert beneficial effects by reducing leukocyte infiltration, downregulating proinflammatory cytokines and free radicals, inhibiting astrocyte activation, decreasing lipid peroxidation and reducing cerebrospinal fluid pressure (Cain and Cidlowski, 2017; Samuel et al., 2017). When given at the recommended neuroprotective doses, however, patients can experience serious adverse side effects such as infections, pneumonia, and gastric bleeding. In fact, several clinical trials have failed to show both safety and efficacy of corticosteroid administration in neurological indications in patients (Bracken et al., 1997; Roberts et al., 2004). We are still far from having a satisfactory drug delivery technology that effectively allows targeting of pathological sites within the CNS without causing undesired toxicity. A better understanding of pathways involved in neuroinflammation can provide new targets that can be exploited in the design of precise and effective drug delivery strategies.

The innate immune response by CNS-resident cells is initially driven by sensing the presence of extracellular or intracellular pathogen-associated molecular patterns (PAMPs) or damage-associated molecular patterns (DAMPs) by pattern recognition receptors (PRRs), such as Toll-like receptors (TLRs) and nuclear oligomerization domain-like receptors (NLRs). Some examples of DAMPs include aggregated proteins characteristic of neurodegenerative diseases, such as β-amyloid, α-synuclein, and microtubule-associated protein-tau; and molecules released from damaged cells, such as ATP, heat shock proteins, oxidized lipids, and chromatin (Thundyil and Lim, 2015). Sensing of DAMPs leads microglia and astrocytes to activate inflammatory signaling pathways, such as NF-κβ, which result in altered cell morphology, increased respiratory metabolism, initiation of phagocytic processes, and release of cytokines, chemokines and reactive oxygen species (ROS) (Burda et al., 2016). Cytokines play crucial roles in cellular communication and neuroinflammatory signaling and can display pro- or anti-inflammatory functions. Important upregulated cytokines in response to alterations in CNS homeostasis are TNF and IL-1β that contribute to recruitment of peripheral immune cells and further activate microglia and astrocytes. Infiltrating neutrophils, macrophages, and lymphocytes sustain and amplify the inflammatory cascade. There is an ongoing conversation focused on clarifying which cells and cytokines have beneficial or detrimental effects in each particular neurological disease. While the initial neuroinflammatory response is thought to be primarily beneficial and reparative, chronic production of proinflammatory cytokines over long periods of time seems to exacerbate tissue dysfunction and degenerative processes (Becher et al., 2017).

A recent focus of research has been the investigation of common neuroinflammation mediators across different neurological conditions. The therapeutic potential of targeting DAMPs, PRRs, and cytokine signaling pathways is being explored in various preclinical animal models. Pharmacological DAMP inhibition has shown therapeutic potential in multiple neurological disorders. High mobility group box 1 protein (HMGB1), for example, is a key protein that initiates neuroinflammation in traumatic, infectious, and neurodegenerative conditions. Preclinical studies have examined HMGB1 modulation by administering small molecule inhibitor compounds or anti-HMGB1 monoclonal antibodies with encouraging results in a wide number of experimental models (Paudel et al., 2018). TLRs are also implicated in several neurological pathologies and have been explored as therapeutic targets to modulate neuroinflammation. TLR4 antagonists, for instance, suppress neuroinflammation by reducing overproduction of inflammatory mediators. These findings were observed in models of neurotrauma, demyelinating disease, neurodegenerative disorders, and viral infections. However, significant adverse effects have been reported (Leitner et al., 2019). Thus, targeting neuroinflammatory mediators might require precise temporal, pathway-targeted interventions adjusted for the unique pathological progression of different neurological diseases.

Current delivery of therapeutic compounds to the CNS have minimal control over targeting specificity and duration of treatment. Systemic high dosages and multiple administration paradigms are often needed to achieve therapeutic concentrations that induce the desired effects in target tissues. Consequently, systemic toxicity and undesired side effects frequently accompany such dosage regimen. Alternative strategies that enhance CNS penetration and targeting include artificial disruption of the blood-CNS barriers (B-CNS-B) and use of direct administration routes. Transient disruption of the B-CNS-B can be achieved by action of chemical or physical agents. Mannitol infusion, for instance, increases cerebral blood flow and vascular permeability, but can also induce toxicity, and provides minimal control over treatment duration and targeting. Alternatively, the use of microbubble-assisted focused ultrasound can induce a more localized and controlled action on the B-CNS-B permeability (Song et al., 2018). Safety of this approach in humans is under evaluation, and clinical application will require precise control over parameters to minimize inflammatory responses, glial cell activation or tissue damage (McMahon et al., 2019). Using direct injection strategies is an alternative option that entails injection of substances directly to the cerebrospinal fluid or CNS parenchyma via intrathecal, intraventricular, or intraparenchymal delivery. Direct injection approaches improve drug exposure, increase local drug concentration, and minimize systemic toxicity (Yi et al., 2014; Pizzo et al., 2018). However, these options are invasive and demand fine and time-consuming technical skills. Additionally, infections, tissue damage, inflammation and drug toxicity often occur after direct drug administration procedures. Therefore, improved strategies that safely and efficiently provide targeted and time-controlled drug delivery to the CNS are needed.

## Properties of Nanomaterials for Drug Delivery

Nanotechnology is defined as the control or restructuring of matter at the atomic and molecular levels, within the range of nano to submicron dimensions (typically 1–100 nm). It involves the manufacturing of nanomaterials, their applications, and integration into physical, chemical, and biological systems (Bhushan, 2017). Advances in nanotechnology for biomedical applications created a variety of nanoparticle-based diagnostic and therapeutic approaches with valuable properties for CNS drug delivery. Delivering therapeutic compounds using nanoparticles improves biodistribution and pharmacokinetics, allows co-delivery of multiple compounds, enables targeted intracellular drug delivery, and reduces systemic toxicity and side effects (Wong et al., 2012). Diverse types of nanocarriers are being explored for drug delivery, including nanoparticles derived from organic (e.g., polyethylene glycol, PEG; poly(lactide-co-glycolide), PLGA), inorganic (e.g., manganese, gold), biological (e.g., lipoproteins, albumin) and hybrid elements (Moeinzadeh and Jabbari, 2017). Among the most widely investigated nanoparticles in drug delivery are liposomes, micelles, dendrimers, and polymeric nanoparticles.

Liposomes are self-assembled lipid bilayer vesicles that can incorporate hydrophilic drugs in the inner aqueous phase, and lipophilic drugs within the phospholipid bilayer. Liposomes are easy to formulate, biocompatible and biodegradable nanoparticles that can be administered via different routes (oral, topical, parenteral) (Torchilin, 2005). Micelles, on the other hand, are hydrophilic shells of amphiphilic block copolymers that typically have smaller diameters (10–100 nm) and allow for controlled release of lipophilic drugs. Both liposomes and micelles are currently used in clinical practice for management of cancer, infections, neurological disease, and other applications (Patra et al., 2018). Limitations in the use of liposomes and micelles include low stability, short shelf life and batch-to-batch reproducibility problems. Overcoming these issues, polymeric nanoparticles introduce control over the molecular architecture, allow precise tuning of drug release, and provide enhanced functionalization capabilities. Dendrimers, for instance, are highly branched and symmetric synthetic nanoparticles whose size, shape, and composition can be accurately controlled (Tomalia et al., 2020). Some interesting properties of dendrimers are size monodispersity, high drug payloads, diverse surface functionalization capability and molecular stability, which make them attractive drug carrier candidates. There is a commercially available antiviral dendrimer formulation in clinical use, and others reported to be in clinical trials for the treatment of solid tumors, and neuroinflammation (Dias et al., 2020). Current limitations with the use of dendrimers involve toxicity associated with positively charged surface groups in higher generation molecules. Other polymeric nanoparticles with simpler architectures, like PLGA, due to their low toxicity profile are FDA-approved for clinical use. Polymer nanoparticles can be derived from natural or synthetic materials. They are generally considered safe, possess flexible loading capacity and are in clinical use in the treatment of cancer, inflammatory and vascular diseases. Polymer nanoparticles are also currently under consideration for several CNS-related applications (Kumar et al., 2020).

As technology advances, sophisticated multifunctional systems are gradually emerging and supplanting the pioneering single purpose first generation nanoparticles. These multifunctional nanoparticles can combine multiple properties in one single system, which has led to the development of theranostic tools. Nanotheranostic tools can incorporate simultaneously diagnostic entities, usually an imaging agent, and therapeutic disease-modifying drugs. For CNS therapeutic applications, nanoparticles that can be visualized using magnetic resonance imaging (MRI), single-photon-emission computed tomography (SPECT), or positron emission tomography (PET) are advantageous. Cahalane et al. (2020) recently described two microglia-targeted polymeric nanoparticles with dual anti-inflammatory and imaging purposes. The authors reported the synthesis of PLGA- and L-tyrosine polyphosphate (LTP)-based nanoparticles loaded with an MRI contrast agent derived from gadolinium, and the anti-inflammatory drug rolipram. Preliminary data suggests preferential uptake by microglial cells and safety *in vitro*. Another recent study proposes the use of theranostic nanoparticles for long-term tracking of transplanted cells coupled with antioxidant treatment following stroke (Yao et al., 2020). Mesoporous core–shell-structured nanoparticles loaded with cobalt protoporphyrin IX and conjugated to ^125^I/spermine-modified dextran polymer conferred protection to transplanted mesenchymal stem cells (MSC) from oxidative stress. Moreover, the imaging properties of these nanoparticles allow MSC tracking to guide intracerebral implantation and evaluate transplanted cell homing. More importantly, the theranostic nanoparticles also promoted angiogenesis, neurogenesis and functional recovery after ischemic stroke in mice.

**FIGURE 1:**
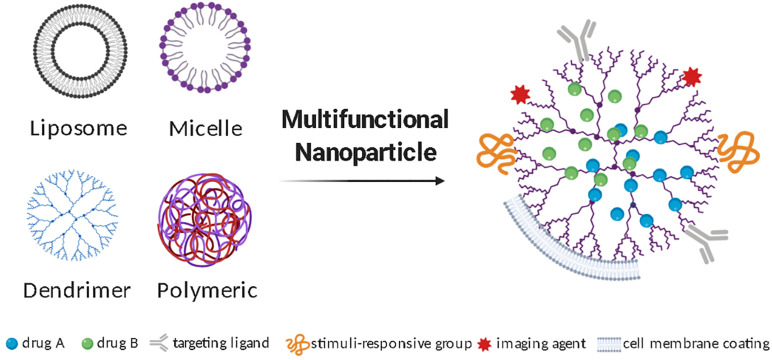
Multifunctional nanoparticles to deliver therapeutics to treat neuroinflammation. Different types of nanoparticles can be used for CNS drug delivery and undergo additional modifications to produce a multifunctional nanoparticle. Illustrative diagrams of the most studied nanoparticle types, as well as some frequently added surface functionalization strategies are represented.

Due to the complexity of CNS pathobiology, combinatorial approaches are likely to be needed in achieving therapeutic efficacy. Multifunctional nanoparticles are amenable to functionalization with addition of surface-grafted biocompatible polymers, cell targeting agents, stimuli-responsive components, energy conversion moieties, or catalytic activities (Figure 1). Addition of cell targeting agents helps direct the nanoparticles to the diseased site increasing bioavailability and exposure. Examples of molecules that have been explored as targeting agents include peptides, antibodies, and aptamers (Patel et al., 2014; Richards et al., 2017). Nanoparticles can also be engineered to adjust their properties in response to external or biological stimuli, such as temperature, light, pH and redox states, and thus control the timing of drug release (Deirram et al., 2019). Redox states and pH, for instance, are altered during CNS inflammation, and can thus be useful stimuli to guide drug delivery from a stimuli-responsive nanoparticle-system carrying anti-inflammatory compounds. Furthermore, nanoparticles can be designed to convert wave energy into heat or chemical energy and thus affect their surroundings. Characteristic applications of these nanoparticles include photothermal and magnetic hyperthermia therapies, as well as focused ultrasound agents (Kim et al., 2018). These recent advances in nanoparticle manufacturing are creating new levels of opportunity in selective biotargeting, also allowing extended retention and drug release in acute and chronic conditions while creating possibilities for remote-controlled drug delivery devices.

### Nanoparticles for Drug Delivery to the CNS

The first preclinical feasibility study evaluating intracerebral injection of liposomes was published in the late 1970s and concluded that safety depends on liposome composition and dose. This work investigated administration of different liposome formulations and doses in rodent models, and opened the possibility of using liposomes as carriers of cell-modifying compounds into diseased CNS tissues (Adams et al., 1977). Since then, over 50,000 peer-reviewed articles have been published reporting new technologies and preclinical evaluations of nanoparticle-based drug delivery for medical applications. About half of these studies are directed to cancer therapies, and only about 1% target neurological conditions, according to a recent PubMed search. Despite a pressing need, there are still insufficient studies investigating the potential of nanoparticle drug delivery for neurological diseases. Even so, there are a few FDA-approved injectable nanoparticles to treat MS (e.g., Copaxone, Plegridy), and an ongoing clinical trial evaluating the potential of using dendrimers as immunomodulators in cerebral adrenoleukodystrophy (clinicaltrial.gov NCT03500627). These are first generation nanomaterials that rely on passive targeting to reach the CNS and accumulate in the pathological site. Understanding how nanoparticle intrinsic properties influence biodistribution and function is an important step in the design of more complex multifunctional nanosystems.

Several studies have explored how physicochemical properties influence nanoparticle penetration and retention in the CNS. There is no golden rule that applies to all nanomaterials, and comparative biodistribution studies are an essential prelude to preclinical studies. Nonetheless, intrinsic nanoparticle properties such as size, shape, and chemical composition influence behavior in biological systems. Furthermore, extrinsic parameters such as surface charge, purity, and colloidal stability are also important determinants of performance. An example is the case of nanoparticles that tend to aggregate when exposed to certain media. Nanoparticle aggregates behave as large particles, with different properties from individual nanoparticles and this can lead to immunogenic effects (Casals et al., 2017). The size of nanoparticles influences biodistribution, and generally smaller nanoparticles penetrate the CNS faster and with higher specificity than larger ones (Mahmoud et al., 2020). In addition to CNS penetration, nanoparticle properties also have an impact on cell targeting (Peviani et al., 2019). For instance, ligand-free 4 nm polyamidoamine dendrimers selectively accumulate in activated microglia and astrocytes in a model of cerebral palsy (Dai et al., 2010). PLGA nanoparticles, on the other hand, are preferentially engulfed by inflammatory monocytes in a model of SCI (Jeong et al., 2017). Interestingly, negatively charged inorganic quantum rods seem to be selectively internalized by neurons (Dante et al., 2017).

In CNS pathologies, different cell types respond distinctively to the same environmental alterations. Thus, achieving high targeting efficiency to specific cell populations can significantly enhance treatments. For instance, during neuroinflammation neurons undergo apoptosis in response to glutamate excitotoxicity. Neuron-targeted dendrimer nanoparticles carrying neuroprotective drugs have shown efficacy in attenuating neurological deficits in a brain injury model (Mishra et al., 2014), supporting the use of nanoparticle-based neuroprotective approaches. Microglia, infiltrating macrophages and astrocytes are key cells involved in the neuroinflammatory response and thus logical therapeutic targets. Several studies have been designed to target microglia and deliver immunomodulatory drugs to attenuate inflammation and reduce tissue damage. Beneficial effects were reported in different models of CNS pathology either by reducing the release of inflammatory mediators or by controlling microglia population at pathological sites (Zhang et al., 2016). Similarly, nanoparticles targeting circulating monocytes/macrophages have confirmed disease-modifying potential in preclinical models of neuroinflammation (Jeong et al., 2017; Han et al., 2019). Although the contribution of astrocytes in neuroinflammation is recognized, nanoparticle strategies to target and modify these cells remain underexplored (Chowdhury et al., 2018).

**FIGURE 2:**
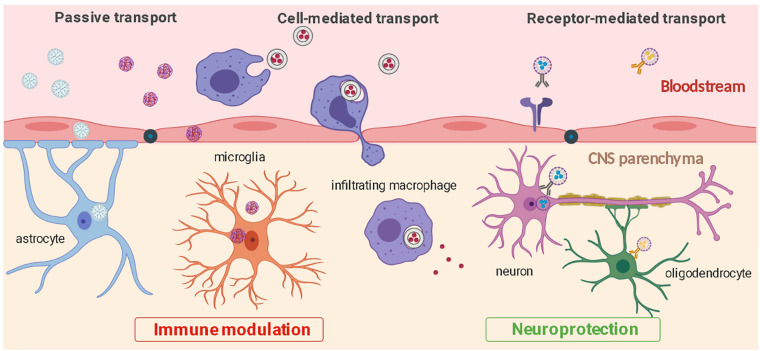
CNS penetration and cell-specific targeting by nanoparticles. Nanoparticles can enter the CNS from the bloodstream through passive transport, ligand receptor-mediated interactions, or through cell-mediated transcytosis. Nanoparticles can be used to efficiently target distinct cell types and modulate inflammation (microglia, astrocytes, infiltrating macrophages) or protect CNS cells from further damage (neurons, oligodendrocytes).

Deeper understanding of neuroinflammatory pathways and CNS cellular biology have led to the development of methods to modify nanoparticle surface with ligands that increase cell specificity and CNS penetration. These include modifications with integrin-binding peptides (Juthani et al., 2020), antibodies (Cerqueira et al., 2012), psychostimulant or psychotropic drugs (Aparicio-Blanco et al., 2019; Saha et al., 2020), B-CNS-B receptor ligands (Hoyos-Ceballos et al., 2020), and neurotropic viruses (Chung et al., 2020). Surface functionalization to improve cell-specificity has been performed on a variety of nanoparticles including liposomes, inorganic nanoparticles, polymeric nanoparticles, and dendrimers (Charabati et al., 2019). Alternatively, magnetic micelles have been reported to enter the brain by application of an external magnet force to the target brain site (Karami et al., 2019). Improved CNS penetrance of nanoparticles can lead to significant improvement in safety and efficacy of pharmacological treatments for neurological diseases. Some of these cell-targeted approaches and nanoparticle transport routes to enter the CNS are illustrated in Figure 2 and summarized in Table 1. In the next sections, we will now review some of the most recent preclinical advances using functionalized nanoparticles for the treatment of neuroinflammation.

**TABLE 1:** Examples of preclinical studies using nanoparticles as cell-targeted drug delivery vehicles to treat CNS pathologies.

Nanoparticle properties	Disease model (species)	Targeted cell/tissue	Therapeutic efficacy	References
**Liposomes**				
Anti-ICAM1/Anti-VCAM TM-mRNA loaded	Acute brain inflammation (mouse)	Endothelial cells	Increased brain penetration; reduced brain edema	Marcos-Contreras et al., 2019, 2020
PTX-loaded	Glioblastoma (mouse)	Neutrophils	Increased brain targeting	Xue et al., 2017
RGD-modified loaded with TFF3	Chronic mild stress (rat)	Monocytes	Increased brain drug concentration, enhanced antidepressant effect	Qin et al., 2015
pH sensitive- DOX loaded	Glioblastoma (mouse)	Tumor cells in acidic environment	Increased anti-tumor and antiangiogenic activity	Zhao et al., 2016
**Micelles**				
PDGF-micelles loaded with TMZ	Glioblastoma (mouse)	PDGFR expressing cells	Selective accumulation in gliomas, reduction of systemic toxicity	Miller et al., 2016
Magnetic naproxen-loaded mPEG-PCL micelles	Healthy (rat)	Remote magnetic field in brain region	Enhanced drug brain penetration, prolonged circulation time	Karami et al., 2019
**Dendrimers**				
NAC/VPA-loaded HO-PAMAM	HCA-induced brain injury (dog)	Microglia, injured neurons	Improved neurological outcomes with lower drug dose; reduced side effects.	Mishra et al., 2014
G4 PAMAM	Cerebral palsy (rabbit)	Activated microglia, astrocytes	Enhanced brain distribution	Dai et al., 2010
**Polymeric**				
PLGA	Spinal cord injury (mouse)	Circulating monocytes	Reduced immune response and scar; improved functional recovery.	Jeong et al., 2017
RBC-CDX coated, DOX loaded PLGA	Glioma (mouse)	Brain endothelial cells	Enhanced brain distribution, improved survival	Chai et al., 2017
NSC-coated PLGA	Ischemic stroke; Traumatic brain injury (mouse)	SDF-1 expressing cells	Enhanced brain delivery, neuroprotection	Ma et al., 2019
RBC-coated NR2B9C-loaded PHB-dextran	Ischemic stroke (rat)	Apoptotic neuronal cells	Reduced infarct area, improved neurological outcomes	Lv et al., 2018
**Other**				
PGP-baicalein loaded solid lipid nanoparticles	Olfactory bulbectomy, depression (mouse)	Neutrophils	Enhanced brain drug concentration, enhanced anti-depressant effect	Chen et al., 2018
Superparamagnetic iron oxide nanoparticles	Acute brain inflammation (mouse)	Monocytes (*ex vivo* loading)	Efficient brain penetration and lentiviral transduction	Tong et al., 2016
Magnetic nanoparticles	Epilepsy (rat)	Monocytes (*ex vivo* loading)	Accumulation on epiloptogenic brain tissue	Han et al., 2019
Neutrophil-derived nanovesicles loaded with RvD2	Ischemic stroke (mouse)	Brain endothelial cells	Diminished neutrophil infiltration and inflammation, increased neuroprotection	Dong et al., 2019

## Cell Targeting Using Nanoparticles to Modulate Neuroinflammation

Active cell targeting can be achieved through nanoparticle modifications that exploit ligand-receptor interaction mechanisms. In the initial stages of neuroinflammation, endothelial cells become activated and play a critical role in immune cell trafficking to the site of inflammation (Ludewig et al., 2019). Upon cytokine stimulation, activated endothelial cells overexpress adhesion molecules, such as vascular cell adhesion molecule-1 (VCAM-1) and intercellular adhesion molecule-1 (ICAM-1) that bind to integrin receptors in circulating immune cells and facilitate their migration into CNS tissue. Nanoparticles conjugated to antibodies that recognize these adhesion molecules in endothelial cells have been developed and investigated in neuroinflammation models. Marcos-Contreras et al. reported that conjugating anti-ICAM-1 or anti-VCAM-1 antibodies to liposomes results in efficient cerebral accumulation of the conjugated-liposomes through active targeting of endothelial cells. Superior penetration rates were measured when compared to transferrin-conjugation, another commonly used strategy to enhance nanoparticle CNS penetration (Marcos-Contreras et al., 2019, 2020). Although CNS targeting was improved, some off-target effects were still detected in these approaches, such as nanoparticle accumulation in the lungs. An alternative strategy to selectively target neuroinflammation sites includes using circulating immune cells as vectors carrying drug-loaded nanoparticles. Neutrophils, lymphocytes, dendritic cells, monocytes, and macrophages can be used as a “Trojan horse” to carry therapeutic formulations in response to CNS inflammation or injury. This is achieved by taking advantage of the intrinsic phagocytic ability of circulating immune cells or by actively targeting these cells conjugating cell-specific ligands to the surface of the nanoparticles.

Several recent studies describe the potential of using peripheral immune cells as carriers of anti-inflammatory nanomaterials to otherwise inaccessible areas of neuroinflammation. Neutrophils are the first immune cells to be recruited to an inflammation site in response to cytokine and chemokine release and remain active in the acute phase of inflammation. Positively-charged lipid nanoparticles loaded with bioactive drugs, such as baicalein or paclitaxel, were shown to be internalized by neutrophils and accumulate in the brains of mice bearing CNS pathology (Xue et al., 2017; Chen et al., 2018). However, neutrophil short lifespan and thus limited therapeutic window limits their use as sustained drug delivery systems for chronic inflammation. Therefore, the potential of using monocytes and macrophages that infiltrate inflammatory sites and remain for longer periods of time is an alternative possibility. Negatively charged liposomes (∼100–200 nm) modified with RGD motifs and loaded with an antidepressant drug were selectively internalized by circulating monocytes after systemic administration. The RGD motif is an arginine-glycin-aspartate peptide sequence that binds to integrin receptors expressed by monocytes. RGD-modified liposomes efficiently targeted the brain and when loaded with an antidepressant drug enhanced behavior in a murine model of depression (Qin et al., 2015). Alternatively, liposomes loaded with clodronate are frequently used as a tool to minimize infiltration of hematogenous macrophages in the context of CNS injury (Popovich et al., 1999). When injected intravenously, clodronate-liposomes induce selective apoptotic cell death of circulating monocytes/macrophages and thus reduce CNS infiltration of these cells. Treatment with clodronate-liposomes promoted functional recovery and reduction of fibrotic scar formation following experimental SCI (Lee et al., 2011; Zhu et al., 2015). In a different approach, Tong et al. loaded superparamagnetic iron oxide nanoparticles into freshly isolated monocytes *ex vivo*. These monocytes were then infused back into a mouse and were detected in areas of neuroinflammation demonstrating the potential to exogenously deliver nanoparticle-carrying monocytes to target inflammation areas in the brain (Tong et al., 2016). A comparable method has also been explored in an epilepsy model where magnetic nanoparticle-carrying monocytes were able to accumulate in epileptogenic brain areas in significantly higher numbers than compared to free nanoparticle administration (Han et al., 2019).

Biomimetic strategies that make use of the intrinsic ability of immune cells to phagocytose foreign materials and target inflammation sites can greatly improve the efficacy of anti-inflammatory treatments using nanoparticle platforms. This approach is applicable in CNS pathologies that involve high grade inflammation and present immune cell recruitment to the CNS. For instance, the use of anti-inflammatory corticosteroid drugs, such as dexamethasone and methylprednisolone, has been supported by a vast collection of preclinical data, which led to more than 150 clinical trials to evaluate clinical efficacy in different CNS pathologies (Sorrells and Sapolsky, 2007). Generally, due to high hydrophobicity there is limited drug availability in CNS tissue, and the recommended systemic corticosteroid doses are high and commonly lead to severe systemic side effects that compromise treatment efficacy (Caruso et al., 2017). Moreover, in conditions where the blood-brain barrier is permeable, a broad range of effects are observed resulting in heterogeneous clinical responses to corticosteroid treatment (Krieger et al., 2014). Incorporating corticosteroids in nanoparticles immediately taken up by circulating immune cells is a promising approach to maximize therapeutic actions of these drugs while minimizing undesired side effects (Cerqueira et al., 2013; Lühder and Reichardt, 2017). Additionally, these strategies have the potential to significantly reduce dosage. Circulating immune cells can be easily obtained from a patient and loaded with drug-loaded nanoparticles that are re-injected into the bloodstream. Limitations associated with using immune cells as nanoparticle carriers relate to accelerated degradation of the nanoparticles within lysosomes, which can decrease drug availability at target sites, and potential undesired alterations in immune cell phenotypes induced by nanoparticles. An illustrative example includes the use of amino-functionalized polystyrene nanoparticles that were shown to perturb mitochondrial function, increase ROS production and trigger inflammasome activation in human macrophages (Lunov et al., 2011). This and other recent studies highlight that nanoparticle internalization can significantly impact macrophage behavior and should be carefully investigated when used for biomedical applications.

### Cell Membrane-Coating Strategies

More recently, the use of cell membrane fragments has been suggested as nanoparticle surface coating material to provide extended circulation times and targeting abilities. Cell membrane-coating strategies offer immune camouflaging, minimize uptake by leukocytes, and can be an attractive option to target non-immune cell types, such as astrocytes and neurons. Decoration of nanoparticles with cell membrane fragments (membrane-coated nanoparticles or backpacks) and production of cell membrane-derived nanoparticles (such as cell-membrane nanovesicles) are emerging methods that provide new diagnostic and therapeutic modalities for different types of diseases (Luk and Zhang, 2015). Membrane-coated nanoparticles are hybrid nanomaterials that display combined properties of biological cell membranes and custom designed synthetic nanoparticles. This technology was initially described to extend the residence time of polymeric nanoparticles *in vivo* by coating them with erythrocyte membranes (Hu et al., 2011). Because erythrocytes are anucleated cells, isolating their membranes is a relatively easy process. Chai and colleagues have designed erythrocyte membrane-coated PEGylated liposomes decorated with a neurotoxin-derived targeting moiety. Doxorubicin-loaded liposomes with these modifications possessed extended circulation times, enhanced therapeutic efficacy, and reduced toxicity in a mouse glioma model (Chai et al., 2017).

Using membrane-coating technologies to produce nanoparticles that mimic the targeting capabilities of immune cells is also being explored. Leukocyte-membrane coating of nanoparticles adds naturally occurring receptor-ligand interactions present in leukocyte membranes, and permits easy passage to inflamed tissues (Parodi et al., 2013). Dong et al. (2019) prepared nanoparticles made of isolated neutrophil membranes and loaded the nanoparticles with therapeutic compounds. The authors reported accumulation of the nanoparticles in ischemic brain areas, reduced inflammation, and improved neurological function proving the feasibility of these approaches (Dong et al., 2019). Cell-membrane nanoparticle coating can be achieved by using virtually any cell type as source of membrane material. More recently, platelets, tumor cells and stem cells have also been explored to construct biomimetic nanoparticles (Zou et al., 2020). PLGA nanoparticles loaded with an anti-edema agent were coated with membranes isolated from neural stem cells (NSC) that have been previously engineered to overexpress CXCR4. CXCR4 is a chemokine receptor for CXCL12 (SDF-1), which is enriched in the ischemic microenvironment. The CXCR4-NSC membrane-coated PLGA nanoparticles accumulated significantly in the ischemic region and enhanced treatment efficacy, by prolonging mice survival and reducing infarct volume (Ma et al., 2019). To further enhance targeting functionality, fusion of membrane material from different cells is being explored. Dehaini et al. (2017) developed hybrid dual-membrane coated PLGA nanoparticles, derived from erythrocytes and platelets, and reported properties of both source cells. When combined functionalities are desired for a single drug delivery nanoplatform, using multiple cell types as source of membrane coating can be a viable option for customizing nanoparticles with added advantages of biocompatibility and refined biological targeting.

In summary, cell-coated nanoparticles offer an additional approach to avoid immune detection, increase sustained circulation, and targeted drug release. Different types of membranes can be leveraged for a range of distinct targeted drug delivery applications without significant biological modifications, which is clinically relevant and can accelerate FDA-approval. The recent advancements in the development of cell-coated nanoparticles capable of sustained drug-release in response to the microenvironment is steadily progressing toward more sophisticated therapeutic strategies and can lead to unique advantages for CNS drug delivery. There is a growing number of patents related to cell membrane coating technologies, which reflects the potential for clinical use (Liu et al., 2019). Although this technology has primarily been explored for cancer therapeutic applications, use in CNS pathologies will likely soon follow.

As naturally occurring nanoparticles in biological systems, exosomes are also gathering interest as therapeutic drug delivery agents. Exosomes are lipid nanovesicles involved in intercellular communication during physiological and pathogenic processes through the transfer of small molecules, such as RNA. After release from their cells of origin, exosomes can be internalized by other cells thereby modulating their function (Zhang et al., 2019). Their stability, biocompatibility, low toxicity, and ability to cross biological barriers make them attractive therapeutic candidates. However, because exosome contents are variable and often poorly characterized, the production of quality-controlled exosomes for clinical purposes is challenging. To overcome these limitations, engineered exosomes loaded with consistent cargoes are being investigated. MSC-derived exosomes tailored to carry high levels of specific miRNAs have shown neuroprotection and improved recovery in rat models of ischemic stroke (Xin et al., 2017). In another study, catalase mRNA delivery by custom-made exosomes attenuated neurotoxicity and neuroinflammation in experimental models of Parkinson’s disease (Kojima et al., 2018). Due to their natural origin and insufficient understanding of the molecular mechanisms associated with their biological action, several challenges still remain in the path to translation to clinical application.

## Stimuli-Responsive Nanoparticles to Treat Neuroinflammation

Another recent focus of research has been the development of nanoparticles that exert therapeutic effects in response to physiological variations or externally applied stimuli. These materials are commonly designated as stimuli-responsive materials, or smart materials, and mimic the responsiveness of living organisms. Smart nanoparticles possess the unique ability to alter their structure in response to slight environmental changes and revert to their original state after the stimulus disappears. Physiological stimuli, such as temperature, pH, redox, oxygen, or enzymes can act as triggers for drug release by causing disruption of covalent bonds between drugs or other molecules and the nanoparticle, or by destabilizing the nanoparticle structure that becomes more permeable for drug diffusion or unveils functional ligands. Stimuli-responsive nanoparticles can thus react not only by releasing therapeutic compounds, but also by exposing and activating surface ligands, such as cell penetrating sequences, targeting ligands, or other functionalities that can alter pharmacokinetics or biodistribution. In addition to the more commonly known pH-sensitive and thermosensitive nanoparticles, many technological advances have allowed materials to be designed to also respond to externally applied stimuli including light, ultrasound, electrical or magnetic fields. pH is a particularly useful stimulus that can be used to control intracellular drug release in response to a changing pH environment from the extracellular medium to endosomes and lysosomes. At the tissue level, it can be used to release drugs in inflammation sites that have typically lower pH levels.

The detailed portrayal and timeline of inflammatory events in neurological diseases can allow the establishment and optimization of precisely timed therapeutic interventions. Administering stimuli-responsive anti-inflammatory nanoparticles can allow precise interventions by only targeting and releasing therapeutic compounds upon detection of microenvironment alterations. In addition to cellular and molecular events, inflammation is also characterized by local microenvironment alterations such as decreased pH, high oxidative stress and accumulation of ROS, and overexpression of matrix-remodeling enzymes (d’Arcy and Tirelli, 2014). pH-responsive nanomaterials have been explored in recent years for drug delivery using nanoparticles that possess pH-sensitive chemical bonds or pH-dependent degradation properties. Redox-sensitive nanomaterials are also available and have potential to be explored in neuroinflammation, either as scavenging agents or drug releasing agents. Enzyme-cleavable nanomaterials can alter functionality in response to the presence of COX, MMP, and other enzymes. Various types of nanoparticles are being designed to possess stimuli-responsive properties, including dendrimers (Wang et al., 2016), liposomes (Lee and Thompson, 2017), micelles (Zhou et al., 2018), and inorganic nanoparticles (Veeranarayanan and Maekawa, 2019).

pH is the most widely studied endogenous stimulus for control of drug release where an acid sensitive spacer between a nanoparticle and cargo is placed (Deirram et al., 2019). Although the use of pH-responsive nanoparticles for drug delivery in neuroinflammatory pathologies remains unexplored, recent research in glioblastoma models validate the use in CNS applications. Most nanoparticles enter cells through endocytic pathways and are, at least momentarily, contained in vesicles during intracellular trafficking. Endosomes and lysosomes have typically lower pH environments (4–5.5) and can thus be used as triggers for cargo release from nanocarriers. Miller et al. designed multifunctional pH-responsive micelles loaded with an anticancer drug to allow for controlled pH-triggered release into glioblastoma cells (Miller et al., 2016). The micelles were prepared with a pH-sensitive lipid to ensure the drug release occurred under acidic conditions. After intravenous injection of the multifunctional pH-sensitive micelles in a mouse model of glioblastoma, selective accumulation in specific brain areas was observed, along with reduction of overall systemic toxicity. In another approach, Zhao et al. (2016) used a pH-responsive and tumor-specific peptide to decorate liposomes containing doxorubicin. The pH-triggered drug release was confirmed *in vivo*, and the anti-tumor activity was increased in mice receiving pH-sensitive liposomes. In addition to triggered drug release in acidic neuroinflammatory environments, pH alterations can also be used as a trigger for intracellular drug release.

Since upregulation of ROS is another feature of neuroinflammation, bioengineered ROS-responsive nanoparticles can be useful in a targeted site-specific drug delivery approach. The responsiveness to intracellular oxidative conditions is achieved by incorporating ROS-labile groups in the nanoparticle, for example boronic ester, proline, or thioketal (Xu et al., 2016). A recent study described the synthesis and evaluation of boronic ester ROS-sensitive nanoparticles, coated with erythrocyte membrane and functionalized with a homing peptide, SHp, that targets ischemic tissue (Lv et al., 2018). This multifunctional smart nanoparticle system was also loaded with the neuroprotective agent NR2B9C. The authors observed prolonged circulation, active targeting to the ischemic site, and drug release in neurons in response to intracellular levels of ROS. Additional serum biochemical analysis and histological evaluation of peripheral organs showed no evidence of toxicity. In another study, Shen et al. (2018) proposed the use of polylactic acid (PLA) as ROS responsive coating for mesoporous silica nanoparticles. The PLA-coated nanoparticles were functionalized with a low-density lipoprotein receptor ligand to enhance CNS penetration, and were loaded with the antioxidant agent resveratrol. *In vitro* studies in models of blood-brain-barrier and inflammatory environments indicated enhanced transcytosis and resveratrol release in response to ROS. These stimuli-responsive nanomaterials can bring important advantages in targeted drug delivery, but they remain largely unexplored in neuroinflammation. Formulating nanocarriers that release drugs only at the target site in response to predetermined signals of disease can have a great impact in the design of effective therapies for neuroinflammation.

## Concluding Remarks

Recent advances in nanoparticle production technologies are creating promising theranostic tools to provide targeted and controlled drug delivery at sites of neuroinflammation. The emergence of biomimetic, cell targeted, and stimuli-responsive multifunctional nanoparticles are among the most encouraging strategies to treat neuroinflammation. Nanotechnology-based approaches remain underexplored for CNS applications, and an expected increase in preclinical studies can accelerate the establishment of nanoparticle-based treatments for CNS pathologies. How can we ensure that this anticipated surge in publications and patents will properly translate into successful clinical applications? Careful consideration of the design, large-scale production ability and standardized characterization of nanoparticles seem essential preludes to ensure adequate conversion into therapeutic products. Systematic approaches to assess the impact of nanoparticle properties on molecular interactions are also critical. Importantly, thorough investigation of toxicity, degradation byproducts and clearance routes of nanoparticles *in vivo* are invaluable in providing safety profiles and promise translatability. Nanoparticles come across as ideal and resourceful tools to provide real time diagnostics and tailored treatment of neuroinflammation and other CNS illnesses, promising to have a significant impact in global health. Continued collaborative efforts between materials scientists, engineers, chemists, neuroscientists, and clinicians can finally identify safe and effective therapies that target neuropathological mechanisms and ultimately mitigate the burden of devastating neurological diseases.

## Author Contributions

SC, NA, and JL conceived the concept and idea of the present review and selected the topics to be discussed. SC did literature searches, read the references, and wrote the first outline of the review. NA and JL reviewed and edited the manuscript. All authors contributed to the article and approved the submitted version.

## Conflict of Interest

The authors declare that the research was conducted in the absence of any commercial or financial relationships that could be construed as a potential conflict of interest.
